# Photosystem II core quenching in desiccated *Leptolyngbya ohadii*

**DOI:** 10.1007/s11120-019-00675-0

**Published:** 2019-09-18

**Authors:** Reza Ranjbar Choubeh, Leeat Bar-Eyal, Yossi Paltiel, Nir Keren, Paul C. Struik, Herbert van Amerongen

**Affiliations:** 1 Laboratory of Biophysics, Wageningen University, Wageningen, The Netherlands; 2 Department of Plant & Environmental Sciences, The Alexander Silberman Institute of Life Sciences, The Hebrew University of Jerusalem, Jerusalem, Israel; 3 Applied Physics Department, The Hebrew University of Jerusalem, Jerusalem, Israel; 4 Centre for Crop Systems Analysis, Wageningen University, Wageningen, The Netherlands; 5 MicroSpectroscopy Research Facility, Wageningen University, Wageningen, The Netherlands

**Keywords:** Photosystem II quenching, Time-resolved fluorescence spectroscopy, Cyanobacteria, Photoprotection

## Abstract

Cyanobacteria living in the harsh environment of the desert have to protect themselves against high light intensity and prevent photodamage. These cyanobacteria are in a desiccated state during the largest part of the day when both temperature and light intensity are high. In the desiccated state, their photosynthetic activity is stopped, whereas upon rehydration the ability to perform photosynthesis is regained. Earlier reports indicate that light-induced excitations in *Leptolyngbya ohadii* are heavily quenched in the desiccated state, because of a loss of structural order of the light-harvesting phycobilisome structures (Bar Eyal et al. in Proc Natl Acad Sci 114:9481, 2017) and via the stably oxidized primary electron donor in photosystem I, namely P700^+^ (Bar Eyal et al. in Biochim Biophys Acta Bioenergy 1847:1267–1273, 2015). In this study, we use picosecond fluorescence experiments to demonstrate that a third protection mechanism exists, in which the core of photosystem II is quenched independently.

**Electronic supplementary material:**

The online version of this article (10.1007/s11120-019-00675-0) contains supplementary material, which is available to authorized users.

## Introduction

Cyanobacteria are photosynthetic organisms which are abundant in aquatic and terrestrial environments. Terrestrial cyanobacteria constitute 28% of total global cyanobacterial biomass and 79% of this is constituted by cyanobacteria living in arid soil crusts (Garcia-Pichel et al. 2003).

Filamentous cyanobacteria live in biological desert soil crusts and face harsh environmental conditions such as high sun light intensity, hydration/rehydration cycles, high daytime, and low nighttime temperatures. These cyanobacteria become desiccated in the hot sunlight and stop their photosynthetic activity. However, they are well adapted to cope with this desiccation and in low light they regain their photosynthetic activity after rehydration (Raanan et al. 2016a, b). High light intensity also challenges these cyanobacteria creating a need for quenching mechanisms to protect the photosynthetic apparatus. Without such protection, reactive oxygen can be produced, which is harmful for the cell. Cyanobacteria have three main photosynthetic antenna systems: phycobilisomes (PBSs), the core of photosystem II (PSII), and the core of photosystem I (PSI). Previous studies have shown that PBSs in the desiccated state lose their organized structure leading to quenching of excitation energy and the loss of efficient energy transfer capacity. Upon rehydration this capacity is regained (Bar Eyal et al. 2015, 2017; Eisenberg et al. 2017). In addition, oxidized PSI was suggested as a quencher of excitation energy in the desiccated state (Bar Eyal et al. 2015).

In this work, we study *Leptolyngbya ohadii,* grown at low light intensities (60 μmol photons m^−2^ s^−1^), by time-resolved fluorescence spectroscopy. We provide evidence that besides quenching of the light-harvesting PBS and PSI, also direct PSII excitation energy quenching occurs in the desiccated state.

## Materials and methods

### Culture growth and sample preparation

The *Leptolyngbya ohadii* cells were grown and prepared in their hydrated and desiccated state as reported in Bar Eyal et al. (2015, 2017). Cultures were incubated in YBG11 medium in shaking flasks (Harel et al. 2004), at 30 °C and 60 μmol photons m^−2^ s^−1^. Cells were homogenized and placed on nitrocellulose filters. The filters were air dried at room temperature for 1 day prior to spectroscopic measurements. The minimal amount of time required for reaching the stable desiccated state was 2 h (Bar Eyal et al. 2017).

### Time-resolved measurements and data analysis

Time-resolved fluorescence of the cells was recorded at room temperature using a picosecond streak-camera system (van Stokkum et al. 2008) as reported before (Bar Eyal et al. 2017; Ranjbar Choubeh et al. 2018), using a time window of 800 ps. The frequency-doubled output of a Ti:sapphire laser (Coherent, Mira) (800 nm) was used to excite the samples at 400 nm. Excitation power was ~ 100 nW (repetition rate 4 MHz) and the spot size was ~ 0.1 mm. The full-width at half-maximum of the instrument response function (IRF) was ~ 8 ps. Data were globally analysed with the use of Glotaran (Snellenburg et al. 2012) and the TIMP package (Mullen and van Stokkum 2007) for the statistical computing and graphics software R. The data from four experiments (four experiments in hydrated state and four experiments in desiccated state) was fitted to a sum of exponential functions convoluted with the IRF (Lakowicz 2006) and the relatively broad IRF determines the shortest time profile that our streak-camera system can measure. A narrow full-width-half-maximum for the Gaussian function correlates with a better time resolution. For our instrument, we could model the IRF as a Gaussian function. The measured fluorescence is obtained as

$${\text{F}}\left( {t,\lambda } \right) = \mathop \int \limits_{0}^{t} {\text{IRF}}\left( {t - x,\lambda } \right) \times \sum\limits_{i} {{\text{DAS}}_{i} \left( \lambda \right) \times \exp \left( {k_{i} x} \right){\text{d}}x},$$

where ${\text{F}}\left( {t,\lambda } \right)$ is the fluorescence, t is the time after excitation, $\lambda$ is the wavelength, $k_{i}$ is the rate by which the *i*th exponential decays, and ${\text{DAS}}_{i} \left( \lambda \right)$ (decay-associated-spectrum) are the amplitudes at the different wavelengths of the *i*th exponential function. *x* indicates time and the integral is performed up to time *t*. By fitting the recorded fluorescence to ${\text{F}}\left( {t,\lambda } \right)$ the rate constants $k_{i}$ and the ${\text{DAS}}_{i}$ are determined. For examples of fit quality, see Figs. S1–S5.

## Results

**Fig. 1:**
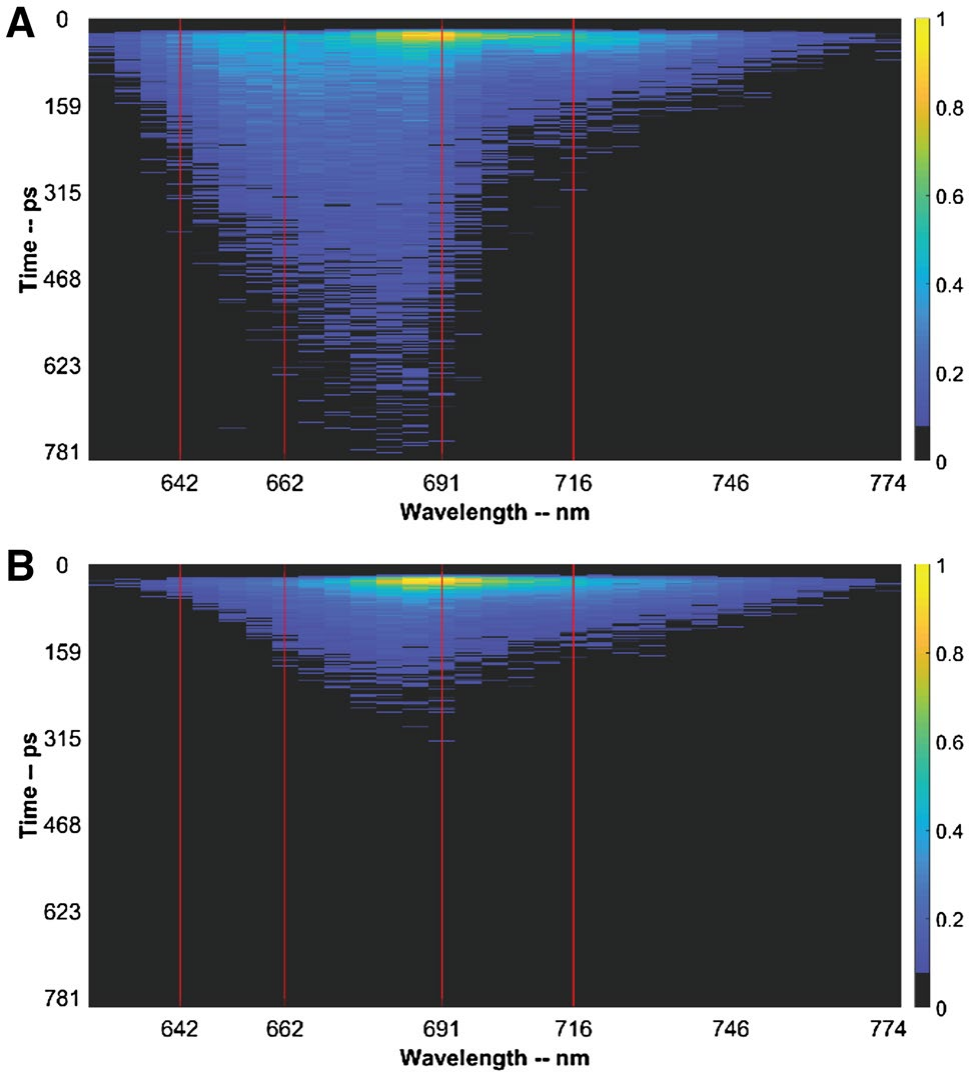
Intensity colour-coded streak-camera images of *L. ohadii* in hydrated (**a**) and desiccated (**b**) state at room temperature. The lifetime of the excited state shortens in the desiccated state. The time traces along the red vertical lines are shown in Fig. 2. The excitation wavelength is 400 nm (mainly exciting Chl *a*)

**Fig. 2:**
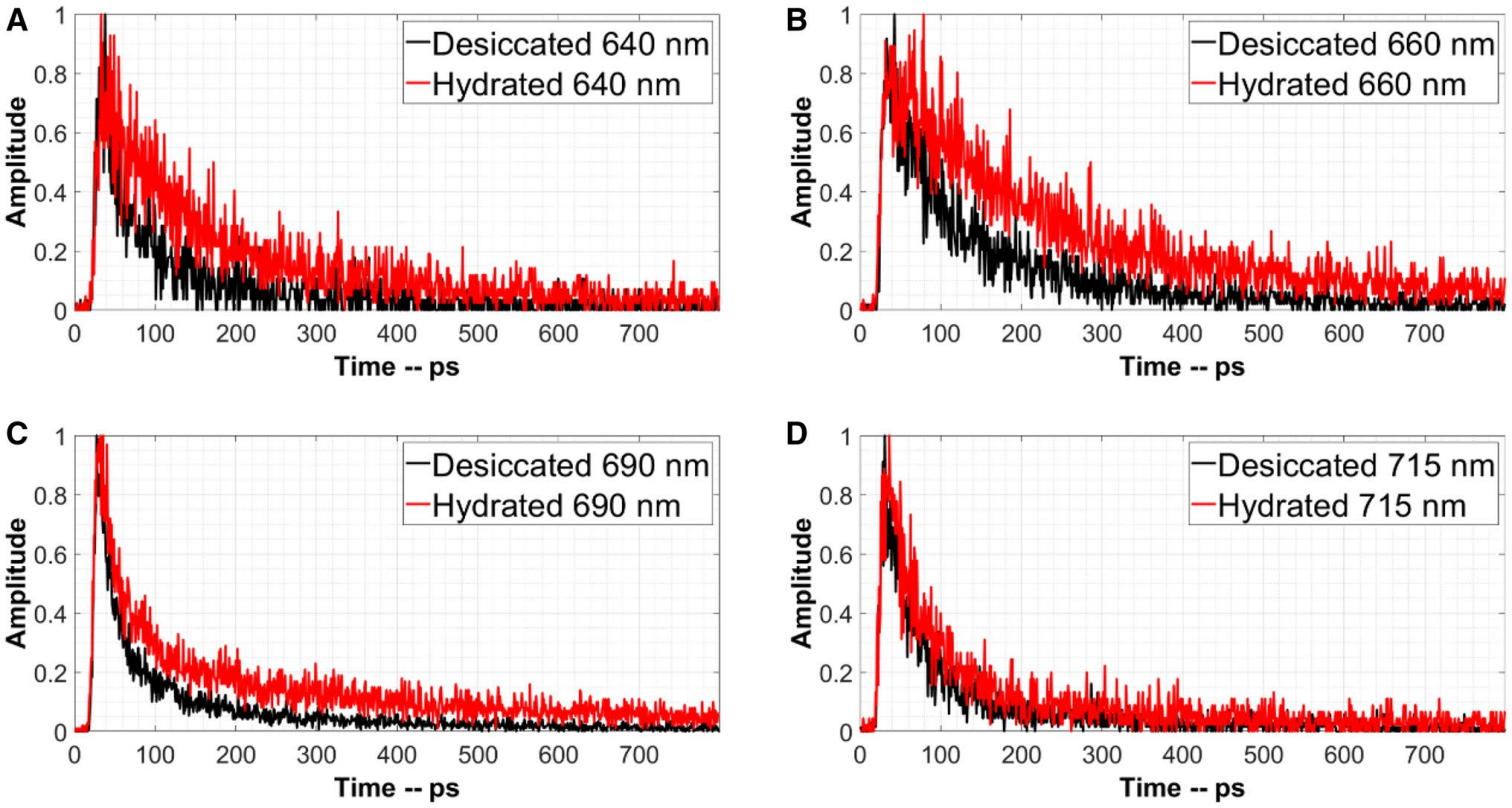
Time traces at the wavelengths marked in Fig. 1 by vertical red lines. In the desiccated state the excited-state lifetime shortens, especially at 690 nm. The excitation wavelength is 400 nm

Figure 1 shows streak-camera images for the hydrated and desiccated states for which an excitation wavelength of 400 nm was used. Light of this wavelength preferentially excites chlorophylls but some phycobilisome excitation is unavoidable. The four red vertical lines mark the wavelengths 640 nm (C-phycocyanin rods (CPC rods)), 660 nm (allophycocyanin (APC) 660), 690 nm (mainly Chlorophyll (Chl) *a* of PSII and some APC680), and 715 nm (Chl *a* of PSI). In the desiccated state, the lifetime of the excited-state population is substantially shorter, especially at 690 nm, which reflects the quenching of excitation energy. The time traces at these wavelengths are shown in Fig. 2.

**Fig. 3:**
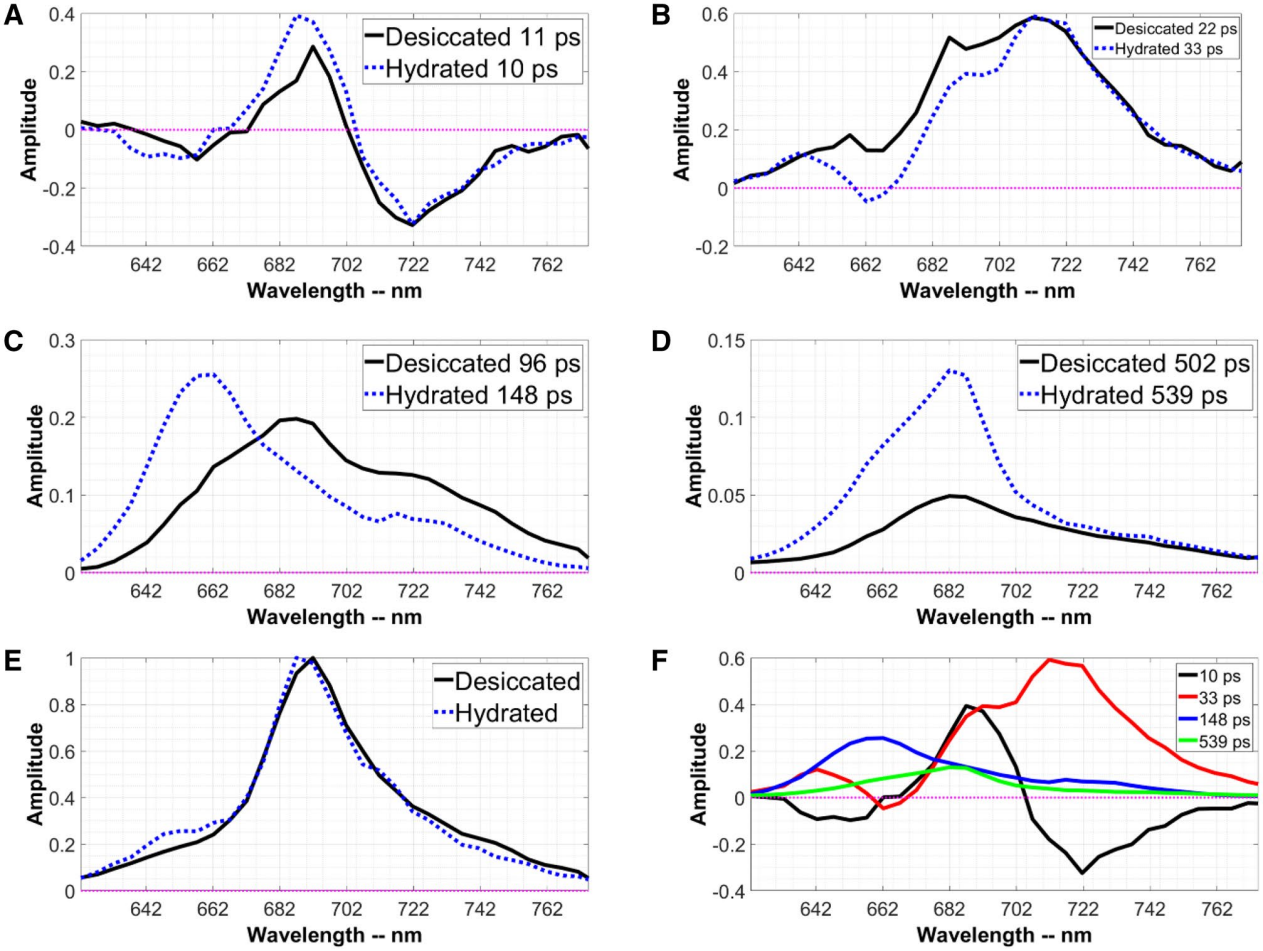
Global analysis of time-resolved fluorescence data of the cyanobacterium *L. ohadii* excited at 400 nm in its desiccated and hydrated state. **a**–**d** The DAS and corresponding lifetimes obtained using global analysis. **e** The time-zero spectrum, which is the fluorescence emission just after excitation and after all ultrafast relaxation processes are finished. The time-zero spectrum was calculated by summing all the DAS in **a**–**d** for each state. **f** All the DAS of *L. ohadii* in the hydrated state to show their relative amplitudes

Figure 3a–d shows the result of a global analysis of time-resolved fluorescence of cells in their desiccated and in their hydrated states. Using the singular value decomposition method (van Stokkum et al. 2004), it was concluded that four lifetimes are required to fit the data for both states. No meaningful fitting was possible with 5 lifetimes. The time-zero spectrum presents the fluorescence emission spectrum directly after excitation assuming that no other relaxation processes occur, and it was calculated by summing all the DAS. This spectrum is shown in Fig. 3e. All the DAS corresponding to the hydrated state are presented in Fig. 3f showing the relative amplitude of different DAS. The fastest component that could be resolved with global analysis has a 10-ps lifetime, both for hydrated and desiccated cells. The corresponding decay-associated spectra (DAS) for both states are given in Fig. 3a. This DAS reflects excitation energy transfer (EET) within the CPC rods of the phycobilisomes (Tian et al. 2011, 2012), manifested by the negative band between 627 and 660 nm. EET within PSI towards long-wavelength Chl *a* is reflected by the combination of a positive band around 690 and a negative one near 720 nm (Tian et al. 2013).

Figure 3b shows 3 positive peaks for both hydrated and desiccated states at 640 nm (CPC rods), 690 nm (mainly Chl *a* and some APC680), and 710 nm (PSI). In the hydrated state, the negative peak at 660 nm corresponds to APC660 and it indicates EET transfer from CPC rods to APC660. A previous study (Bar Eyal et al. 2017) showed that the excitation energy is quenched within CPC rods in the desiccated state, which is reflected by the positive peak at 660 nm in Fig. 3b. Note that in the hydrated state in which EET is not quenched the amplitude in Fig. 3b at 660 nm is negative which indicates EET. The lack of EET to APC660 in the desiccated state shows up as a lack of negative amplitude around 660 nm. The amplitude of the DAS in the PSI emission region (> 700 nm) is the same for the desiccated and the hydrated state; however, the fluorescence lifetime is shorter in the desiccated state (22 ps) than in the hydrated state (33 ps), which demonstrates that also PSI is being quenched, in agreement with the data presented before (Bar Eyal et al. 2015).

**Fig. 4:**
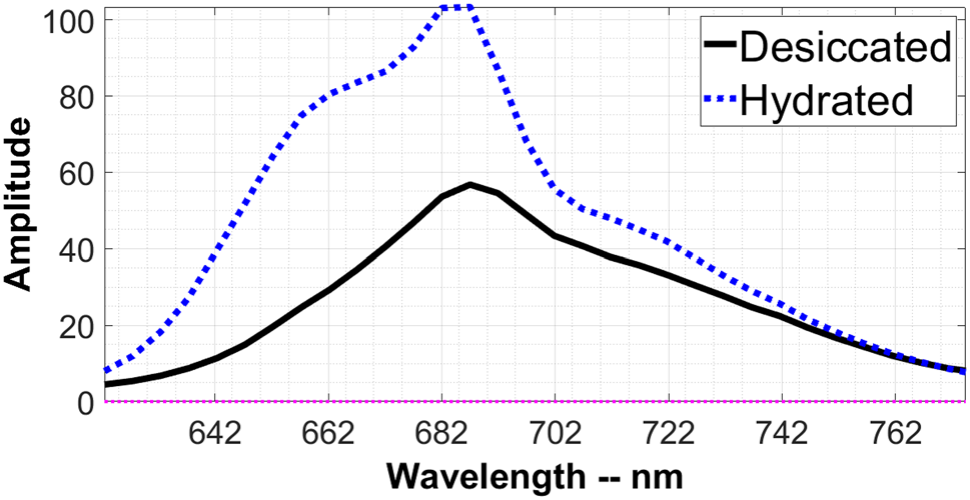
The calculated steady-state spectra of *L. ohadii* in both the desiccated and hydrated state. The fluorescence is substantially quenched in the desiccated state at 640–660 nm (CPC rods and APC660), 690 nm (mainly Chl *a* of PSII and some APC680), and to a lesser extent at 715 nm (Chl *a* of PSI)

Figure 3c shows the next DAS for the desiccated and the hydrated states, both corresponding to a 98-ps lifetime. The APC660 peak at 660 nm, which is prominently present in the hydrated state, is substantially reduced in the desiccated state. This is due to the lack of EET from CPC to APC660 in the desiccated state as was already clear from Fig. 3b. The peaks at 690 nm and 720 nm belong to PSII and PSI, respectively, which have an increased amplitude in the desiccated state. The increase in amplitude is accompanied by a concomitant decrease in amplitude in the same wavelength region of the long-lifetime component, as shown in Fig. 3d. This increase in amplitude of the short-lived component at the expense of the amplitude of the long-lived component largely reflects the fluorescence quenching. This phenomenon can also be observed in the calculated steady-state spectrum shown in Fig. 4 as a reduction in the fluorescence amplitude.

Figure 3d shows 3 bands around 660 nm (APC660), 690 nm (mainly Chl *a* of PSII and some APC680), and 720 nm (PSI). The peak at 660 nm is substantially reduced in the desiccated state, because the excitation energy does not reach APC660 and is quenched in the CPC rods as reported previously (Bar Eyal et al. 2017). Also, the peaks of PSII and PSI at 690 nm and 720 nm are reduced in the desiccated state. This reduction is accompanied by an increase of the amplitude in the DAS of Fig. 3b and c. Both results indicate that PSI and PSII are also directly quenched in the desiccated state. The change in the amplitude of the PSII emission is greater than that of PSI, which shows PSII is quenched to a greater extent than PSI.

These experiments were also performed in the presence of a saturating concentration of DCMU, a PSII inhibitor that binds to the Q_B_ site and inhibits photochemical energy transfer (see Fig. S6). The differences observed between hydrated and desiccated states were similar, demonstrating that the effect is not photochemical in nature.

## Discussion

Desert-dwelling cyanobacteria need to protect themselves against high light intensity during the day, which in their natural habitat is accompanied by drought. It was reported before that these cyanobacteria do this by quenching the excitation energy within the CPC rods of the PBSs (Bar Eyal et al. 2017) and PSI Chl *a* molecules (Bar Eyal et al. 2015). The results in the present study confirm those findings but, in addition, suggest that the core of PSII is also quenched in the desiccated state. This finding matches a pattern that is observed in different types of organisms. It was observed before that in diatoms part of the antenna system from the PSII core uncouples in high light conditions, while both the uncoupled antenna and the PSII core become quenched separately (Miloslavina et al. 2009; Chukhutsina et al. 2014). A similar pattern has been proposed for photoprotective non-photochemical quenching (NPQ) in plants by Holzwarth and coworkers [for an overview see (Jahns and Holzwarth 2012)] in which fast quenching is ascribed to the detachment of light-harvesting complexes accompanied by their aggregation and quenching, followed by quenching of the PSII core plus attached antenna complexes on a slower time scale. Both for diatoms and plants, this quenching of the core plus attached antenna complexes is thought to relate to the involvement of carotenoids via a xanthophyll cycle, but such a cycle is absent in cyanobacteria.

Among the desiccating organisms, lichens seem to make use of two quenching mechanisms, one is operating at the level of the antenna and the other, which was attributed to spill-over from PSII to PSI, is operating at the level of the PSII core (Slavov et al. 2013). In principle, spill-over might contribute to the quenching of PSII in *L. ohadii*. This might be detected via the disappearance of PSII emission and the concomitant appearance of PSI emission. However, we do not find any convincing proof for that. The increase of the fluorescence above 700 nm in Fig. 3b in the desiccated state is due to a rise in PSII emission, whereas the peak of PSI at 720 nm is more or less the same for the desiccated and hydrated states. This is reminiscent of the situation in the cyanobacterium *Synechococcus elongatus* 7942 in which quenching of the PSII core was observed upon going from state I to state II. Also in that case spill-over could be ruled out because no such change in PSI emission was observed (Ranjbar Choubeh et al. 2018). The most likely explanation for quenching of the PSII core is non-radiative energy dissipation within its reaction centre. This has been suggested as a highly effective protective mechanism against photodamage upon excessive excitation in cyanobacteria before (Sane et al. 2002; Cser and Vass 2007). Furthermore, the possibility of non-radiative excitation energy quenching occurring within the reaction centres of PSII and its functional/physiological implications have been discussed for various photosynthetic organisms (Ivanov et al. 2003, 2008). Similar conclusions have been reported in a number of studies on desiccated lichens, and they all emphasize the role of an auxiliary quenching mechanism within the PSII reaction centre (Heber 2008; Heber et al. 2006a, b, 2007, 2011).

In conclusion, like many other photosynthetic organisms, also *L. ohadii* makes use of multiple photoprotective quenching mechanisms. In this particular organism, one leads to the quenching of the antenna and two others cause the quenching of the cores of PSI and PSII, respectively. However, the underlying physical model for the novel mechanism described here remains to be elucidated.

## Electronic supplementary material

Below is the link to the electronic supplementary material.

Supplementary material 1 (DOCX 2595 kb)
