# Supplementary material for: Photosystem II core quenching in desiccated Leptolyngbya ohadii
Source: Photosynth Res. 2019 Sep 18;143(1):13–8. doi: 10.1007/s11120-019-00675-0 (PMC6930311; doi:10.1007/s11120-019-00675-0)
Supplement: Supplementary file 1 — Supplementary material 1 (DOCX 2595 kb) [file 11120_2019_675_MOESM1_ESM.docx]

**Photosystem II core quenching in desiccated *Leptolyngbya ohadii***

Reza Ranjbar Choubeh^a^, Leeat Bar Eyal^b^, Yossi Paltiel^c^, Nir Keren^b^, Paul C. Struik^d^, Herbert van Amerongen^a,e^

^a^Laboratory of Biophysics, Wageningen University, Wageningen, the Netherlands

^b^Department of Plant & Environmental Sciences, The Alexander Silberman Institute of Life Sciences, The Hebrew University of Jerusalem, Jerusalem, Israel

^c^Applied Physics Department, The Hebrew University of Jerusalem, Jerusalem, Israel

^d^Centre for Crop Systems Analysis, Wageningen University, Wageningen, the Netherlands

^e^MicroSpectroscopy Research Facility, Wageningen University, Wageningen, the Netherlands


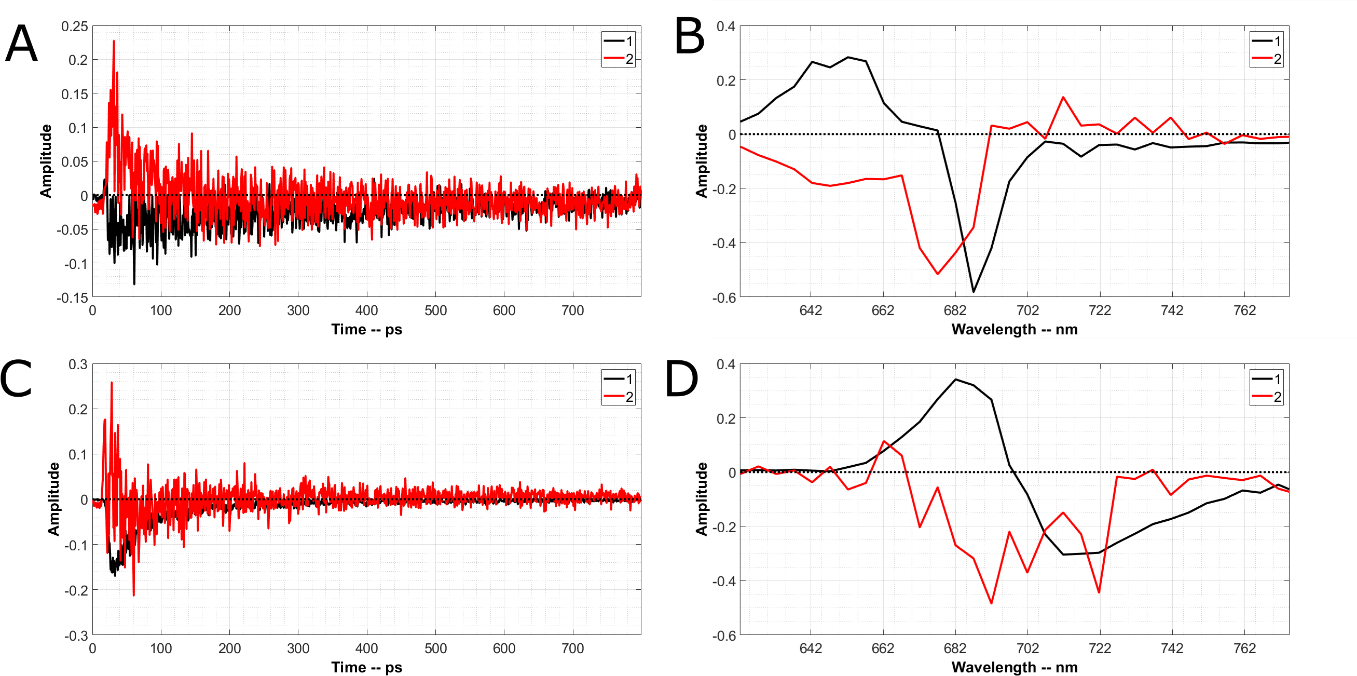


Figure S1. The first two left singular (A and C) and right singular (B and D) vectors of the residual matrix are shown. The left singular vector indicates the quality of fit for the obtained lifetimes and the right singular vector indicates the quality of fit for the obtained decay-associated spectra (DAS). Figures A-B belong to the hydrated data and Figures C-D belong to the desiccated data for which their global analysis is presented in Figure 3. The randomness of these vectors indicates a good quality of fit. For example, if the data could have been fitted with an extra exponential function, in the left singular vector of the residual matrix, a decay curve would be observed. The slight deviation from zero for left and right singular vectors is due to fitting multiple data sets with the same lifetimes. For each hydrated and desiccated state, the data from four measurements was used in the global analysis. Figure S1 belongs to one data from each hydrated and desiccated state. Other data sets have similar plots.


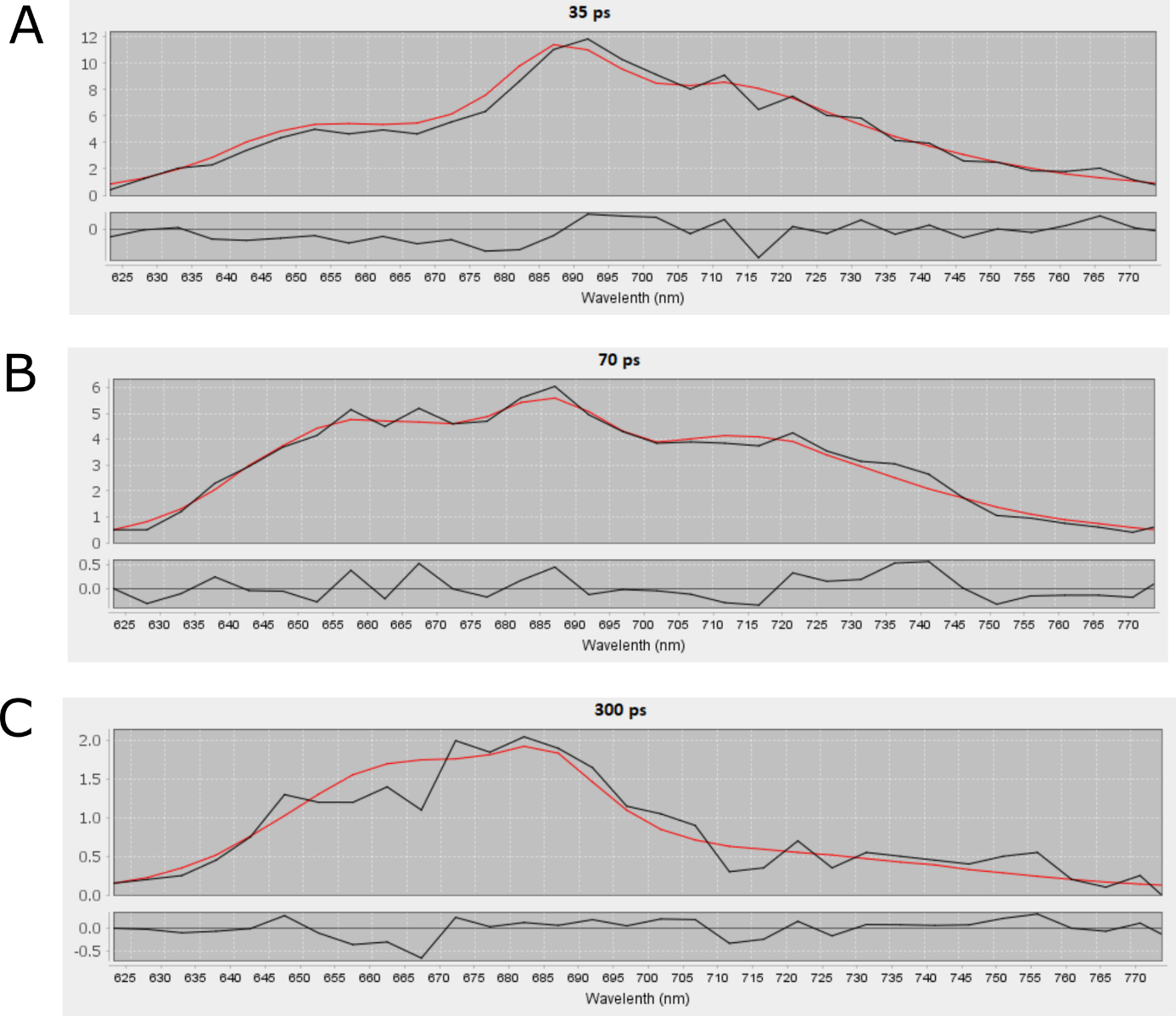


Figure S2. The raw fluorescence data and the fitted amplitude at different times after excitation are shown. Each chosen time point has a width of ~ 1 ps. Below each figure the difference between the fit and data is plotted. The data belongs to the hydrated data for which its global analysis is shown in Figure 3. For hydrated state the data from four measurements was used in the global analysis. Figure S2 belongs to one data out of the four measurements. Other data sets have similar plots.


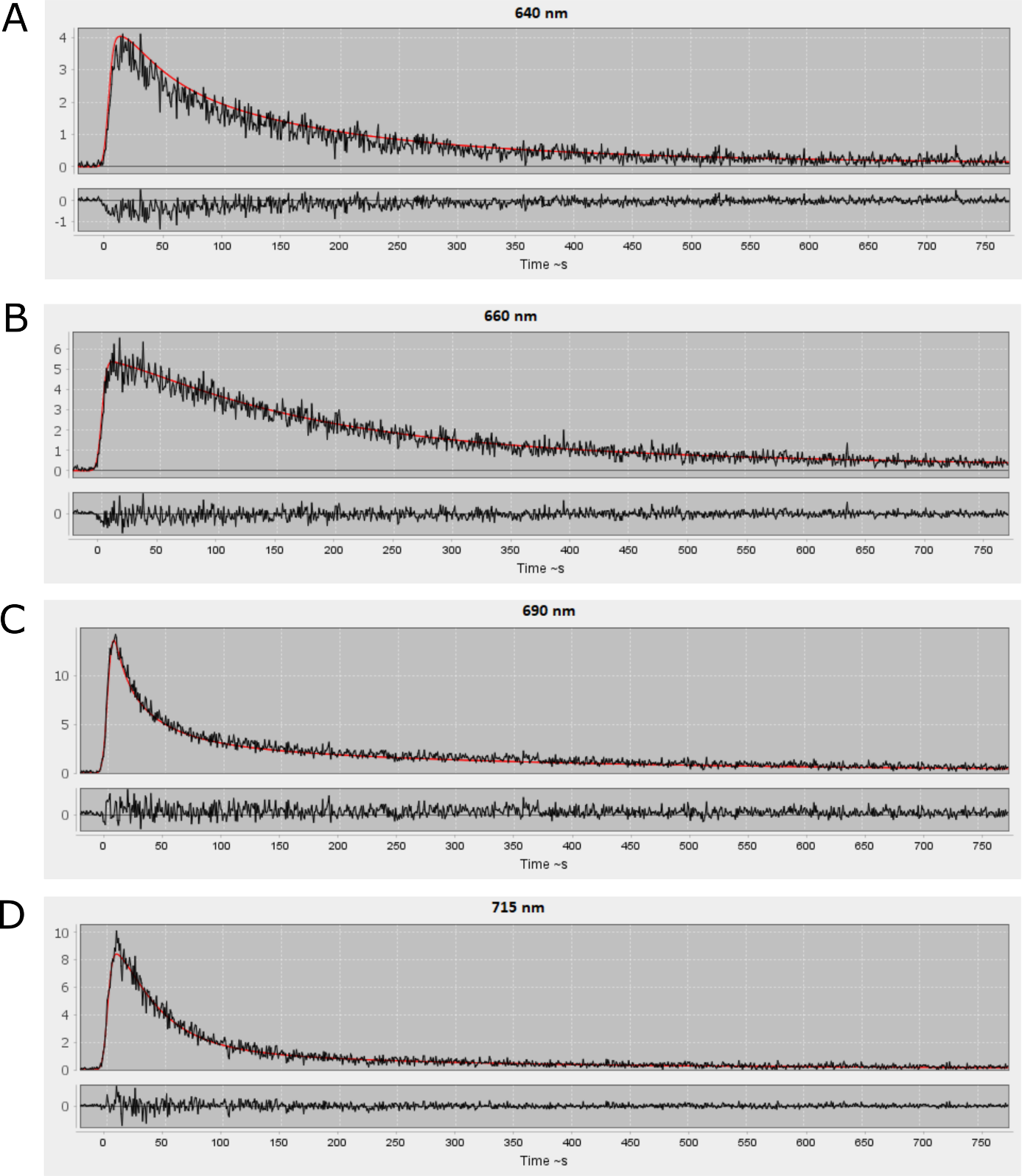


Figure S3. The raw data and the fitted time traces at four different wavelengths are shown. Each chosen wavelength has a width of ~5 nm. Below each figure the difference between the fit and data is plotted. The data belongs to the hydrated data for which its global analysis is shown in Figure 3. For hydrated state the data from four measurements was used in the global analysis. Figure S3 belongs to one data out of the four measurements. Other data sets have similar plots.


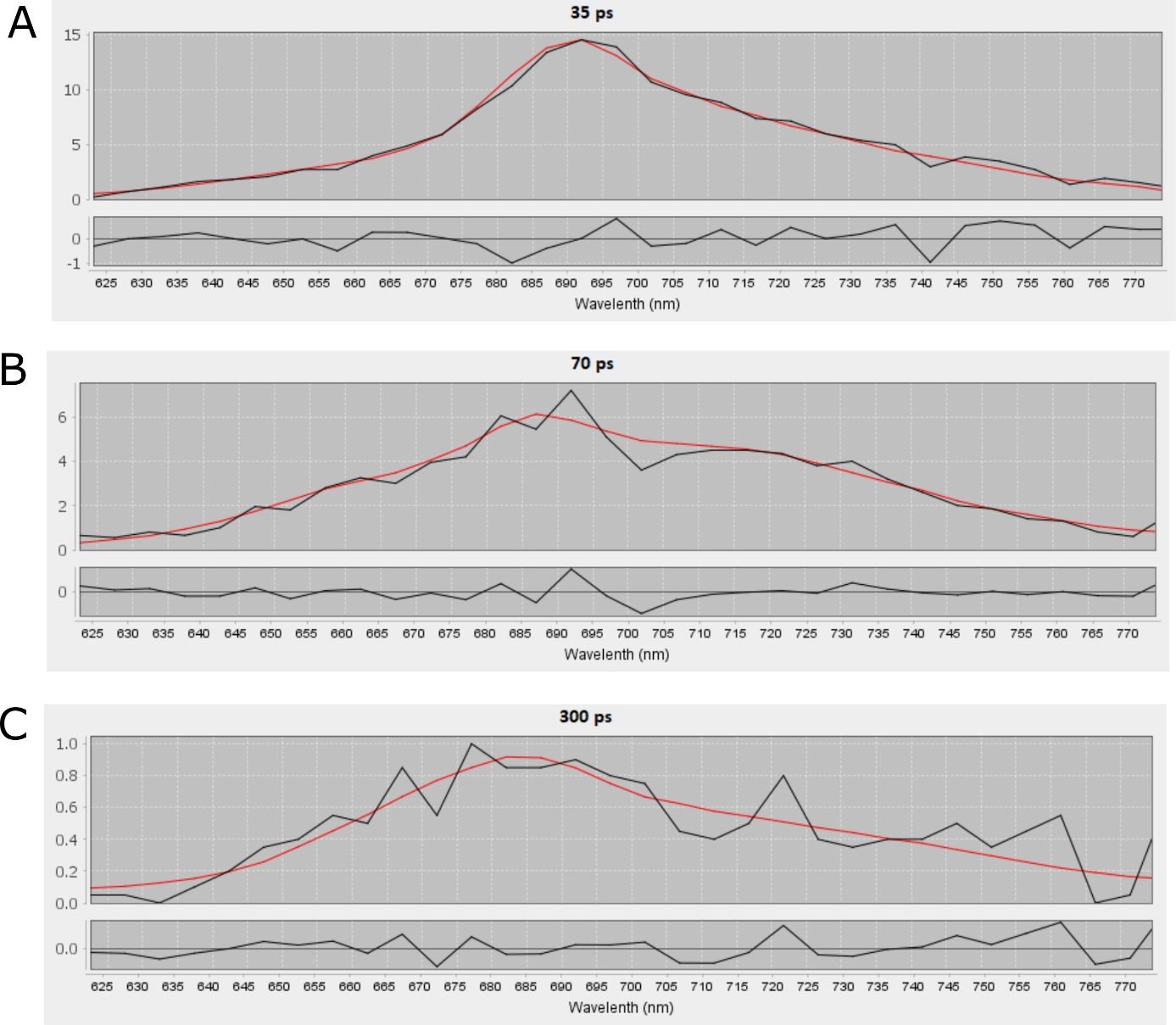


Figure S4. The raw fluorescence data and the fitted amplitude at different times after excitation are shown. Each chosen time point has a width of ~ 1 ps. Below each figure the difference between the fit and data is plotted. The data belongs to the desiccated data for which its global analysis is shown in Figure 3. For desiccated state the data from four measurements was used in the global analysis. Figure S4 belongs to one data out of the four measurements. Other data sets have similar plots.


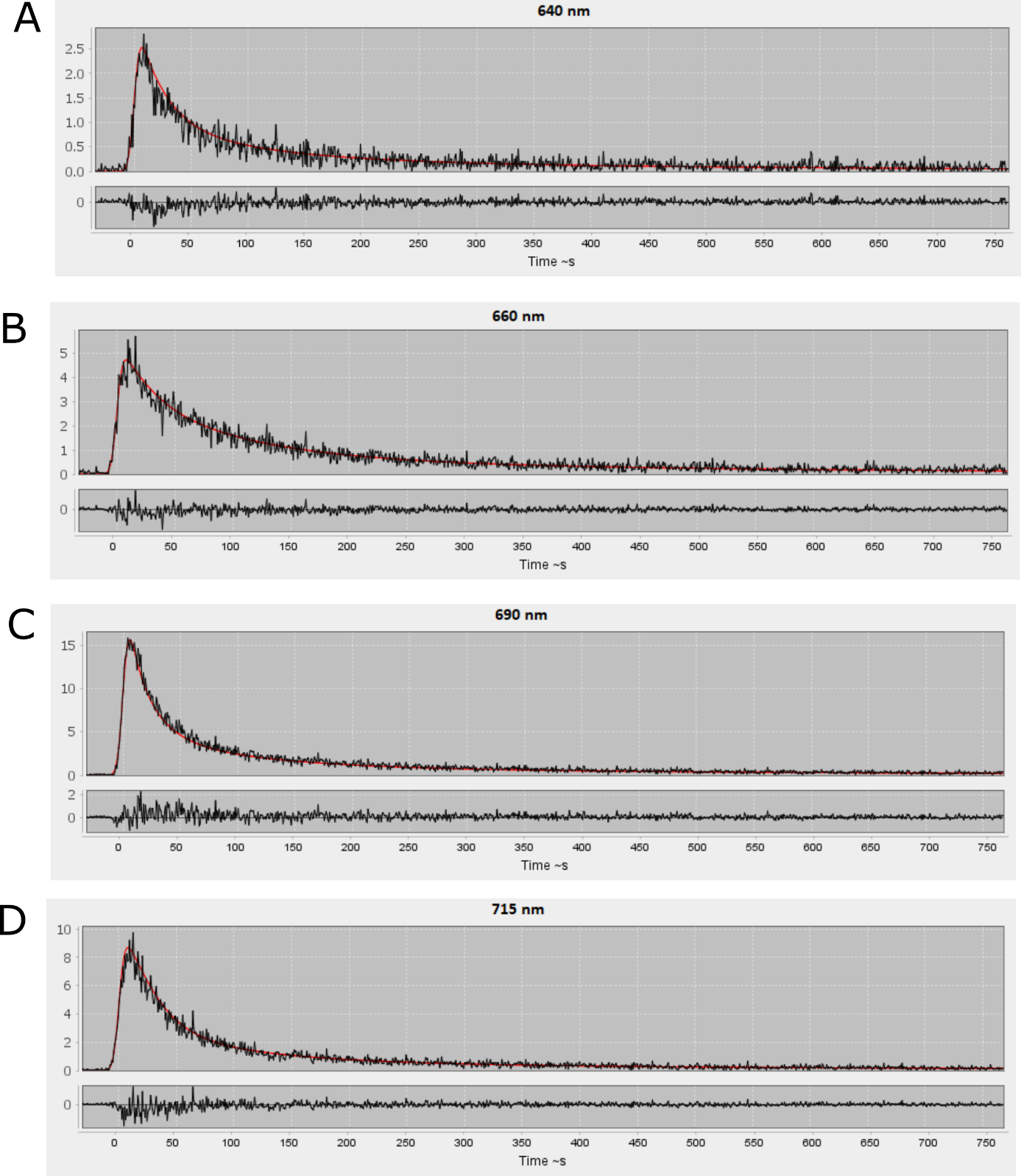


Figure S5. The raw data and the fitted time traces at four different wavelengths are shown. Each chosen wavelength has a width of ~5 nm. Below each figure the difference between the fit and data is plotted. The data belongs to the desiccated data for which its global analysis is shown in Figure 3. For desiccated state the data from four measurements was used in the global analysis. Figure S5 belongs to one data out of the four measurements. Other data sets have similar plots.


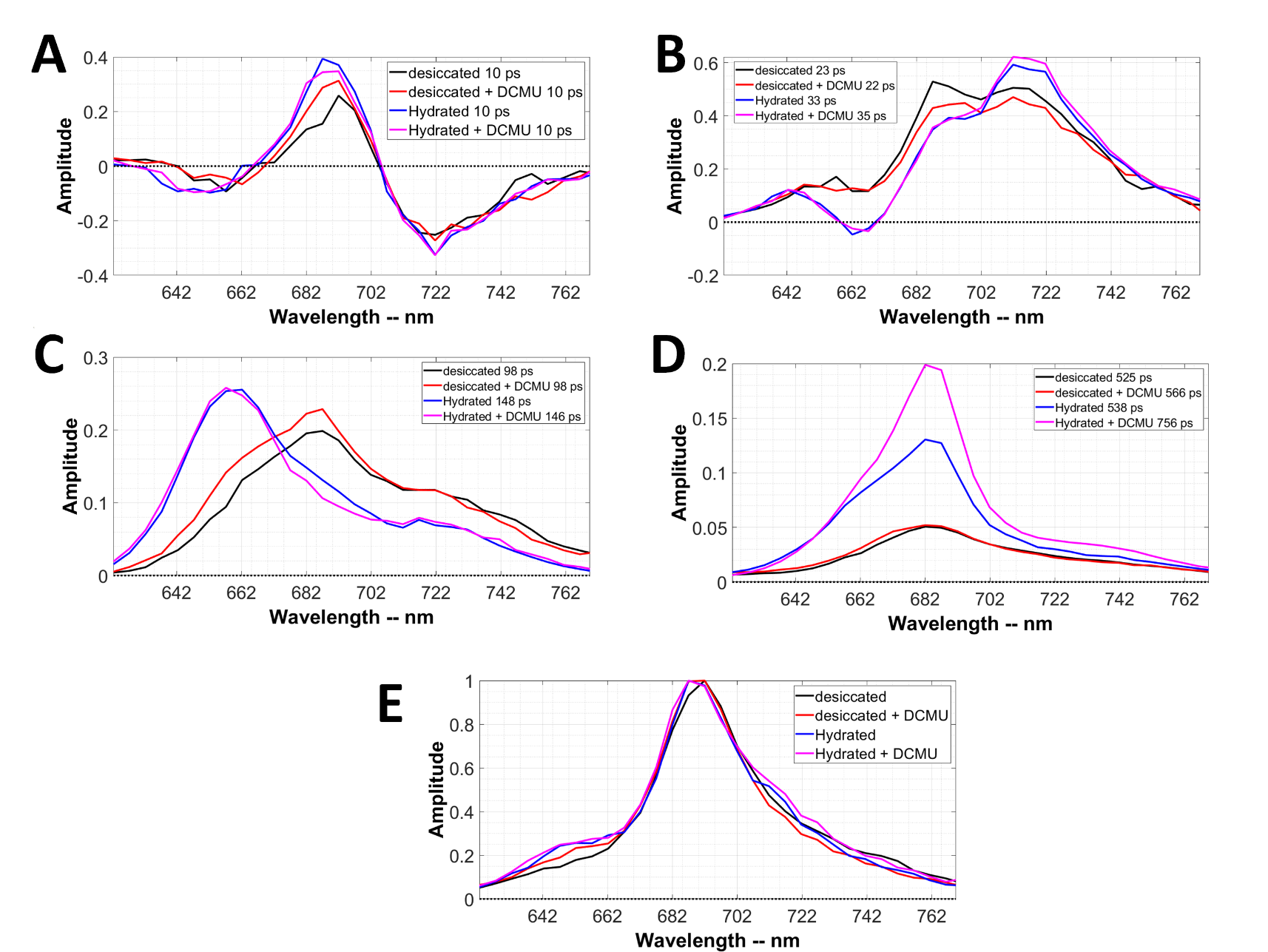


Figure S6. The Leptolyngbya ohadii’s decay-associated spectra (DAS) obtained by global analysis of time-resolved fluorescence is shown in A-D. The time-zero spectrum (the sum of all DAS showing the fluorescence emission just after excitation and after all ultrafast relaxation processes are finished) is shown in E. The DAS belongs to cells in desiccated and hydrated states in presence and absence of 10 μM DCMU. The excitation wavelength is 400 nm.
